# Circulating Biomarkers of Interstitial Lung Disease in Systemic Sclerosis

**DOI:** 10.1155/2012/121439

**Published:** 2012-09-03

**Authors:** Harpreet K. Lota, Elisabetta A. Renzoni

**Affiliations:** Interstitial Lung Disease Unit, Royal Brompton Hospital, London, SW3 6NP, UK

## Abstract

Interstitial lung disease (ILD) is a major cause of morbidity and mortality in patients with systemic sclerosis (SSc). Although a large proportion of SSc patients have only limited interstitial involvement with an indolent course, in a significant minority ILD is progressive, requiring prompt treatment and careful monitoring. One of the main challenges for the clinician treating this highly variable disease is the early identification of patients at risk of progressive ILD, while avoiding potentially toxic treatments in those whose disease is inherently stable. Easily available and repeatable biomarkers that allow estimation of the risk of ILD progression and early response to treatment are highly desirable. In this paper, we review the evidence for circulating biomarkers with potential roles in diagnosis, monitoring of disease activity, or determining prognosis. Peripheral blood biomarkers offer the advantages of being readily obtained, non-invasive, and serially monitored. Several possible candidates have emerged from studies performed so far, including SP-D, KL-6, and CCL18. Presently however, there are few prospective studies evaluating the predictive ability of prospective biomarkers after adjustment for disease severity. Future carefully designed, prospective studies of well characterised patients with ILD, with optimal definition of disease severity and outcome measures are needed.

## 1. Introduction 

**Figure 1:**
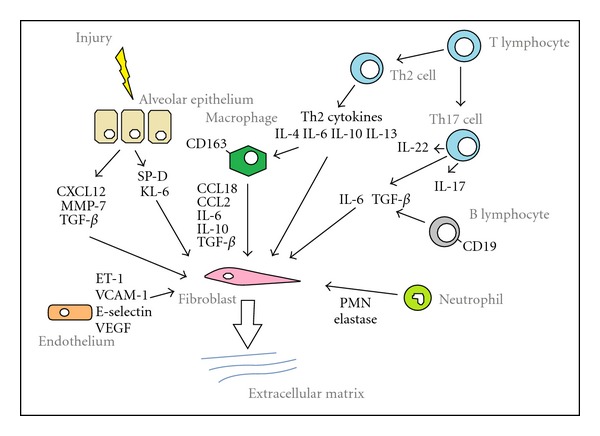
Potential biomarkers in SSc-ILD.

Systemic sclerosis (SSc) is a multisystem, autoimmune connective tissue disease, characterised by excessive extracellular matrix deposition, with remarkable heterogeneity in organ involvement pattern and prognosis. Pulmonary involvement, due to pulmonary fibrosis or pulmonary hypertension, is the leading cause of mortality [1, 2]. The pathogenesis of pulmonary fibrosis in SSc involves a complex combination of epithelial and endothelial cell injury with inflammatory and immune activation. Occurring in response to unknown initiating factors, the interaction between vascular, epithelial, and immune dysfunction leads to dysregulated fibroblast activation and increased extracellular matrix production [3]. This paper will focus on the circulating biomarkers for SSc-associated interstitial lung disease (SSc-ILD), as summarised in Figure 1.

A degree of interstitial involvement is present in the majority of patients with SSc, although severity of lung disease at presentation and subsequent longitudinal behaviour are highly variable. In view of the marked variability in the natural history of SSc-ILD, markers of the likelihood of ILD progression are needed in clinical management. Patients with a recent diagnosis of SSc are more at risk of ILD progression, as the rate of forced vital capacity (FVC) decline is highest during the first five years since diagnosis [4]. The severity of ILD also has clear prognostic implications. Decreased FVC and diffusing capacity of the lung for carbon monoxide (DLCO) have been repeatedly identified as risk factors for progression, as has the extent of ILD on CT [5–7]. Recently, Goh et al. have proposed a simple staging system which subgroups SSc-ILD into limited and extensive, based on rapid estimation of CT extent, integrated, if necessary, by FVC levels. This has been found to be more accurate prognostically than either CT or FVC in isolation [8] and can be easily applied in the clinical setting to provide rapid estimation of likelihood of ILD progression. By contrast, bronchoalveolar lavage (BAL) findings provide only limited prognostic information and are not linked to long-term survival or the rapidity of progression of lung disease [9, 10], although may provide insights into pathogenetic mechanisms. Histological pattern is also not linked to likelihood of disease progression. In contrast to the idiopathic setting, a pattern of usual interstitial pneumonitis (UIP), seen only in a minority of patients, is not associated with a significantly worse survival than the nonspecific pneumonitis (NSIP) pattern, by far the most common [11]. Contrary to previous perceptions, ILD is found almost as frequently in limited compared to diffuse skin disease, and the rate of FVC decline does not appear to differ between the two subsets [12]. 

ILD in the context of SSc can be asymptomatic and patients may first present with extensive lung disease. On the other hand, most patients with SSc have limited ILD involvement, which will have a stable course even without treatment. The reliable detection of patients at risk of lung disease progression is particularly difficult in patients with mild, early disease. The identification of non-invasive, prognostic biomarkers that predict the likelihood of disease progression has the potential to allow timely immunosuppressant therapy, while avoiding unnecessary treatment in patients likely to follow an indolent course. Furthermore, identification of patients more at risk of disease progression is needed for cohort enrichment in randomised controlled clinical trials, which have so far included a proportion of patients with stable SSc-ILD, thus potentially diluting the effects of the intervention [13]. 

Awareness of potential confounding factors is crucial to correctly interpret biomarker associations with SSc-ILD. In the context of SSc, serum biomarkers can reflect extra-pulmonary disease activity, including rapidly progressive diffuse skin fibrosis and active systemic disease. From the pulmonary aspect, DLCO is a sensitive marker of ILD, but also reflects pulmonary vasculopathy. Indeed, a disproportionate reduction in DLCO in relation to lung volumes should prompt the assessment of pulmonary hypertension [14]. Adjustment for these factors in multivariate analysis is crucial to assess the correlation between the putative biomarkers and SSc-ILD.

Biomarkers can fulfil a number of roles, including identification of pathways involved in disease pathogenesis, assessment of disease severity, prediction of future disease behaviour and use as surrogate outcome measures [15]. This paper focuses on peripheral blood biomarkers, as they offer a number of advantages—they are readily obtained, can be measured longitudinally, and thus have the potential to be integrated into clinical use.

## 2. Autoantibody Subsets

Autoantibody subsets have strong associations with different patterns of pulmonary involvement. Anti-topoisomerase I antibodies (ATA), have been consistently associated with pulmonary fibrosis, while anti-centromere antibodies (ACA) are linked to pulmonary hypertension and are rarely present in SSc-ILD [16]. While it is clear that ATA positivity is associated with a greater risk of lung fibrosis, it remains unclear whether it is associated with more progressive ILD. A reduced incidence of lung fibrosis is found in anti-RNA polymerase III positive patients [17]. The nucleolar autoantibodies, anti-U3 RNP antibody and anti TH/To, are linked to an increased risk of pulmonary disease and appear to be associated with development of pulmonary hypertension disproportionate to the degree of interstitial involvement [18].

## 3. Alveolar Epithelial Proteins 

Although the sequence of events in the pathogenesis of SSc-ILD is not fully understood, ultrastructural studies have shown that both epithelial and endothelial cell injury may precede inflammation and fibrosis [19]. The increased clearance of technetium-labeled diethylene triamine pentaacetate (99mTc-DTPA) aerosol from the lungs of patients with SSc-ILD is indicative of a breached epithelial barrier and is associated with more rapid decline in FVC [20, 21], independently of disease severity. Lung epithelium-specific proteins leaking into the circulation may thus represent potential biomarkers of alveolar epithelial damage.

### 3.1. Surfactant Proteins (SP-A and SP-D)

Secreted by type II alveolar epithelial cells and airway Clara cells, pulmonary surfactants are lipoprotein complexes that include the hydrophobic proteins SP-B and SP-C and hydrophilic proteins SP-A and SP-D [22]. In addition to stabilising alveolar surface tension, they play an important role in the lung's host defence system, a role mediated primarily by SP-A and SP-D. 

Serum levels of SP-A and SP-D are significantly higher in SSc patients with pulmonary fibrosis than in those without [23, 24], and SP-D serum levels are negatively correlated with VC and DLCO [24, 25]. SP-D levels are more sensitive than SP-A in detecting ILD as defined by CT. Using cut-off levels set at 43.8 ng/mL for SP-A and 110 ng/mL for SP-D, the sensitivities and specificities for detecting CT-positive ILD in 42 patients with SSc were 33% and 100% for SP-A and 77% and 83% for SP-D, respectively [23]. Receiver operator curve analysis demonstrated good sensitivity (89.4%) and specificity (80%) of SP-D levels >90 ng/mL in the assessment of “alveolitis” as defined by BAL neutrophilia and/or HRCT ground glass in the Scleroderma Lung Study [26]. In a small but prospective study of 35 patients with SSc-ILD followed over 1–10 years, SP-D levels were seen to definitely increase over time in 9 out of the 10 patients with worsening ILD, as defined by changes in symptoms, lung function, and imaging, compared to mild increases in only 3 out of 25 patients with stable or improving SSc-ILD [24]. In a small, retrospective study of 6 patients with SSc-ILD by Yanaba et al., serum SP-D levels were analysed over a mean follow-up period of 2.3 years. In 3 out of the 4 patients treated with immunosuppressive therapy, longitudinal SP-D levels decreased/stabilised in parallel with lung function findings [25]. 

### 3.2. Krebs Von Den Lungen 6 (KL-6)

KL-6 is a high-molecular-weight mucin-like glycoprotein, strongly expressed by type II alveolar epithelial cells and bronchiolar epithelial cells [27], which increases following cellular injury and/or regeneration. Additionally, KL-6 has been shown to have profibrotic and antiapoptotic effects on lung fibroblasts and thus may have a role in the pathogenesis of SSc-ILD [28]. 

Serum KL-6 is elevated in a variety of different ILDs, including the idiopathic interstitial pneumonias, hypersensitivity pneumonitis, drug-induced pneumonitis, sarcoidosis, and connective tissue disease-associated ILD (CTD-ILD), reflecting the prominent role of alveolar epithelial injury and proliferation across ILDs [29]. Serum levels of KL-6 are significantly higher in SSc patients with pulmonary fibrosis than in SSc alone, and levels inversely correlate with VC and DLCO [25, 30]. A threshold of 500 U/mL, commonly used also in the context of other interstitial lung diseases, provided a 78.8% sensitivity and 90% specificity in detecting “alveolitis” as defined in the Scleroderma Lung Study [26]. In a study of 240 patients with a variety of CTDs, a serum level >500 U/mL was a marker of ILD (sensitivity 0.79; specificity 0.93; diagnostic accuracy 0.896), and a level >1000 U/mL was a marker of “active” progressive ILD (sensitivity 0.867; specificity 0.865; diagnostic accuracy 0.866), although this was loosely defined as disease requiring newly added intervention [31]. Bonella et al. found a stronger correlation for KL-6 than SP-D with HRCT fibrosis scores and confirmed the described association with FVC and DLCO [32].

In a retrospective longitudinal study, KL-6 levels were determined in 39 patients with SSc during a follow-up period of 0.3–6.1 years [33]. New onset or deterioration of ILD occurred in 4 patients and was associated with a rapid increase in serum KL-6 levels. Reflecting its potential use in serial monitoring of disease activity, levels of KL-6 have been shown to decrease after treatment with cyclophosphamide [34]. Satoh et al. evaluated the prognostic significance of serum KL-6 levels in 219 patients with ILD, including 67 with CTD-ILD. Higher levels of KL-6 were seen in patients who died during the follow-up period, with a threshold level of 1000 U/mL discriminating nonsurvivors from survivors [35].

 Overall, KL-6 appears to be a promising multipurpose biomarker that appears to reflect response to treatment and prognosis in SSc-ILD. Although integrated into clinical practice in Japan for a number of years, it still requires validation in a large number of patients with SSc-ILD, particularly to assess whether it can predict outcome independently of markers of ILD severity. 

## 4. CC and CXC Chemokines 

Chemokines traffic leucocytes to sites of inflammation and are classified into subsets based on the position of their first two cysteine residues: CC and CXC chemokines. Both bind to receptors expressed on the surface of leucocytes, CXCR and CCR, antagonists of which may represent candidates for targeted therapy.

### 4.1. CC Chemokine Ligand 18 (CCL18)

CCL18, previously known as pulmonary and activation-regulated chemokine (PARC), is constitutively expressed at high levels in the lungs, mainly by alveolar macrophages, and acts as a chemoattractant for a variety of mononuclear cells. CCL18 production by alveolar macrophages, alternatively activated by Th2 cytokines, is increased in a number of fibrotic lung diseases, including SSc-ILD, and correlates with serum levels [36]. CCL18 has been shown to stimulate fibroblast collagen synthesis [37], which in turn appears to further increase CCL18 production [38]. 

In SSc, serum CCL18 levels are inversely correlated with baseline lung function and have been associated with longitudinal changes in VC and DLCO [36, 39]. In a longitudinal analysis of 21 patients with SSc-ILD by Kodera et al., serum CCL18 correlated with ILD activity (determined by HRCT, lung function, and BAL analysis), possibly more tightly than KL-6 or SP-D. In a recent, prospective cohort study of 83 SSc patients over a four-year period by Tiev et al., increased serum CCL18 levels were independently predictive of ILD worsening [40]. A baseline CCL18 level >187 ng/mL was the best cut-off (53% sensitivity; 96% specificity) for identifying subsequent lung function worsening (10% decrease from baseline of TLC or FVC) or death, with a hazard ratio of 5.36 (95% CI 2.44–11.75; *P* = 0.001), even after adjustment for baseline DLCO and duration of disease [40]. To our knowledge this is the first large study to prospectively evaluate the prognostic ability of a biomarker in SSc-ILD, adjusting for ILD severity and other covariates. Interestingly, CCL18 has also been prospectively evaluated in patients with idiopathic pulmonary fibrosis by Prasse et al. and was found to predict early lung function decline and mortality, with a similar cut-off level of 150 ng/mL, again independently of disease severity [41], suggesting its utility as a marker of ILD progression independently of the setting. Overall, if confirmed by separate prospective studies, these results suggest that serum CCL18 could be used in clinical management as a marker of progressive ILD, to aid in targeting treatment to the correct patients, and should be evaluated as a potential therapeutic target.

### 4.2. CC Chemokine Ligand 2 (CCL2)

CCL2, also known as monocyte chemoattractant protein-1 (MCP-1), is chemoattractant for monocytes and T cells and has been shown to induce Th2 cell polarisation, thereby stimulating collagen production and myofibroblast differentiation in fibroblasts [42, 43]. Serum levels are upregulated in SSc and correlate with the presence of ILD [44–46]. In a study of 33 SSc patients, serum CCL2 variation correlated with changes in VC during a 3-year follow-up period [47], although the number of patients with declining lung function was small. Bronchoalveolar lavage CCL2 concentration was associated with the presence of ILD in 32 SSc patients and correlated with lung function parameters and CT scores [48].

### 4.3. CXC Chemokine Ligand 10 (CXCL10)

CXCL10 displays strong chemoattractant activity for Th1 lymphocytes with elevated serum levels seen in various autoimmune diseases [49]. In SSc, serum levels are significantly increased in the presence of ILD compared to those without and normal controls [46, 50]. However, a recent retrospective, longitudinal study of 31 SSc patients found that levels did not correlate with change in lung function over time [47]. 

### 4.4. CXC Chemokine Ligand 12 (CXCL12)

The expression of CXCL12 and its receptor CXCR4 is critical for the recruitment of circulating progenitor cells during tissue repair. Circulating CXCR4+ progenitor cells have been observed to correlate with skin and lung involvement [51] and are found in the lung tissue of patients with SSc-ILD but not in controls, with parallel upregulation of its ligand CXCL12, expressed by alveolar epithelial cells and alveolar macrophages [52].

## 5. Leukocytes 

### 5.1. T-Cell Subsets

Two functional subsets of T cells with distinct cytokine-secretion profiles are well recognised [53]. Type 1 (Th1) T cells predominantly produce interleukin-2 (IL-2) and interferon-gamma (IFN-*γ*), whereas type 2 (Th2) T cells produce interleukins IL-4, IL-5, IL-6, IL-10, and IL-13 [54]. These subsets appear to exert opposing roles in tissue remodelling and fibrogenesis; IFN-*γ* suppresses fibroblast activity whereas the Th2 cytokines such as IL-4, IL-6, and IL-13 are profibrotic either by directly stimulating collagen synthesis by fibroblasts or indirectly by inducing profibrotic cytokines such as transforming growth factor-beta (TGF-*β*) and connective tissue growth factor (CTGF) [55].

In patients with SSc, a predominant Th2 cytokine profile has been reported in lung tissue [56], bronchoalveolar lavage fluid [57], and peripheral blood [56]. In SSc-ILD, a further marked reduction in the Th1/Th2 ratio compared to SSc patients without ILD has been reported, with a strong linear correlation between a lower Th1/Th2 ratio and lower FVC levels [56]. Serum levels of IL-10 and IL-6 have been found to be elevated in patients with SSc-ILD compared to SSc alone [47, 58], and our group has recently shown that elevated IL-6 is independently associated with lung function worsening and increased mortality in patients with SSc-ILD (submitted for publication). Serum levels of IL-15, a pleiotropic cytokine which may have a pathogenetic role in both fibrotic and vascular lung disease, were found to strongly correlate with impaired lung function in SSc [59].

Th17 lymphocytes, recently described as a subset which synthesise an array of cytokines including IL-17A, are the main producers of TGF-*β* among Th subsets [60]. Increased Th17 cells and IL-17A levels have been detected in the sera, skin, and lungs of patients with SSc [61–63], although another study reported normal serum IL-17A levels in SSc [64]. Th17 is induced by various cytokines including IL-23, IL-6, and TGF-*β*; interestingly, IL-23 has also been associated with the presence of ILD in SSc [65]. IL-22 is a cytokine which plays a role in the maintenance and integrity of epithelial barrier function [66], produced by Th17 and Th22 lymphocytes, another novel T-cell subset [67]. Circulating IL-22- and IL-17-producing T cells were increased in SSc individuals presenting with ILD, as detected by HRCT and decreased TLC, compared with those without ILD [68].

### 5.2. B Cells

B-cell infiltration has been recently demonstrated in SSc lung and skin [69, 70]. There is evidence of circulating B cell activation in SSc [71] with overexpression of CD19, a positive regulator that increases B-cell signals in response to antigens [72]. In patients with SSc-ILD, B-cell-depleting therapy with Rituximab significantly improved lung function in a small, randomised, controlled study by Daoussis et al. [73], suggesting a crucial role of B-cells in the progression of fibrosis in SSc. These results suggest circulating B-cell markers should be assessed in relation to lung involvement, as they may ultimately identify subsets more likely to respond to B-cell depletion treatment strategies. 

## 6. Macrophages/Monocytes

Macrophages, mostly derived from CD14+ monocytes, can display two distinct phenotypes of activation: Th1 or “classically” activated (M1) macrophages arise in response to IFN-*γ* or IL-1 whereas Th2, or “alternatively” activated (M2), macrophages are derived following exposure to Th2 cytokines, including IL-4, IL-13, and IL-10 [74], and are characterised by an anti-inflammatory and profibrotic phenotype. The CD14+ monocyte fraction from peripheral blood in patients with SSc-ILD is characterised by substantially increased expression of the activation marker CD163, which colocalises with the M2 marker CD204 [75], and, following stimulation with lipopolysaccharide, release of the profibrotic mediators CCL18 and IL-10 [76], characteristic products of M2 macrophages. There also exists a population of CD14+/CD45+/collagen I-producing circulating monocytes which are increased in the peripheral circulation of patients with SSc-ILD compared to controls [76].

## 7. Matrix Metalloproteinases/Tissue Inhibitors of Metalloproteinases

Remodelling of the extracellular matrix and maintenance of basement membrane integrity involve a balance between the matrix metalloproteinases (MMPs) and their inhibitors, tissue inhibitors of metalloproteinases (TIMPs). MMP-7 (matrilysin), a metalloproteinase which targets a broad range of extracellular matrix proteins, was originally found to be highly overexpressed in IPF lungs [77] and subsequently in other interstitial lung diseases [78]. In IPF, both BAL and serum MMP-7 are negatively correlated with FVC and DLCO [79]. Among SSc patients, higher levels of serum MMP-7 were seen in patients with lung fibrosis compared to those without and were associated with lower DLCO levels, although the association of MMP-7 levels with ILD progression was not evaluated [80]. Notably, patients with lung fibrosis and concomitant pulmonary hypertension had higher mean MMP-7 serum levels compared to those with lung fibrosis alone, indicative of the potential confounding issue of underlying vasculopathy in DLCO reduction. 

MMP-9 (gelatinase B), associated with chronic inflammatory autoimmune diseases and conditions characterised by excessive fibrosis, was significantly increased in serum of SSc patients compared to healthy controls [81] and in bronchoalveolar lavage of SSc-ILD patients [82]. Interestingly, circulating levels of MMP-12 have been reported to be tightly inversely correlated with FVC (*r* = −0.82), and MMP-12 staining to be significantly increased in SSc-ILD lungs compared to normal controls [83]. TIMP-1 levels have been shown to correlate with the presence of ILD in SSc and to negatively correlate with DLCO, albeit weakly (*r* = −0.28) [84]. 

## 8. Neutrophil Elastase

Polymorphonuclear neutrophilic leukocyte (PMN) elastase is a serine proteinase that is believed to modulate extracellular matrix formation and remodelling following lung injury [85]. As elegantly reviewed by Hant and Silver, serum levels of PMN elastase have been reported as significantly increased in SSc-ILD and interestingly correlated well with SP-D and KL-6 [15, 86]. 

## 9. Profibrotic Growth Factors

Serum connective tissue growth factor (CTGF) has been found to be increased in SSc patients, and to correlate with skin and lung fibrosis [87, 88], although longitudinal studies to evaluate prognostic ability have not been performed. Interestingly, serum TGF-*β*, considered one of the master regulators of tissue fibrosis, is not consistently upregulated in SSc patients and indeed was found to be reduced in patients with active diffuse skin disease, perhaps reflecting sequestration to active SSc skin disease [89].

## 10. Markers of Oxidative Stress

Oxidative stress mediated by free oxygen radicals is believed to play a role in the pathogenesis of lung fibrosis and of systemic sclerosis. Serum total antioxidant power, a measure of global antioxidant activity, was found to be increased in SSc patients, although did not differ according to presence of ILD [90]. By contrast, serum isoprostane, a marker of lipid peroxidation, was associated with SSc-ILD and was inversely correlated with FVC and DLCO (*r* = −0.4 for both) [91]. Urinary levels of F2-isoprostanes, generated by free radical peroxidation of arachidonic acid, also inversely correlated with DLCO (*r* = −0.44). Although the association with ILD severity is interesting, none of the oxidative stress markers were assessed as potential predictors of ILD progression. 

## 11. Acute-Phase Proteins 

Acute-phase reactant proteins are elevated in a proportion of patients with SSc. An elevated erythrocyte sedimentation rate (ESR) has been shown to be independently associated with mortality in a number of studies [92, 93]. C-reactive protein (CRP) significantly correlates with ESR [94] and with serum IL-6 in SSc [95]. In 1043 SSc patients, CRP was observed to correlate with disease severity, poor pulmonary function, and shorter survival [94]. Sharing a C-terminal pentraxin domain with CRP, pentraxin 3 (PTX3), an acute-phase protein produced at disease sites by a number of cell types, including fibroblasts [96], was found to correlate with lung function impairment in SSc [97]. 

## 12. Vitamin D

Insufficient levels of vitamin D have been reported in a number of autoimmune diseases [98], including SSc [99]. In a study of 327 patients with SSc, vitamin D deficiency was associated with reduced DLCO (*P* < 0.02) [100]. An association with DLCO and/or increased pulmonary artery pressures on echocardiography had also been reported in two smaller earlier studies [99, 101], suggesting a possible correlation with pulmonary vasculopathy, rather than ILD.

## 13. Endothelial Cell Activation

Ultrastructural studies have shown that both epithelial and endothelial cell injury precede inflammation and fibrosis [19]. Following endothelial activation, a procoagulant and profibrotic environment ensues. Endothelin-1 (ET-1), an endothelial cell product with well-known vasoconstrictor properties, has also been shown to promote profibrotic processes, including the induction of myofibroblast differentiation, contraction, and extracellular matrix synthesis [102]. Increased ET-1 levels have been found in BAL fluid and lung tissue from patients with SSc [103, 104]. Increased serum levels of soluble vascular cell adhesion molecule-1 (sVCAM-1), soluble E-selectin (sE-selectin), vascular endothelial growth factor (VEGF), and endothelin-1 (ET-1) have been described in patients with SSc, and have been variably correlated with internal organ involvement, including pulmonary fibrosis [105–107]. Anti-endothelial cell antibodies (AECA), postulated to play a role in vascular injury in a number of autoimmune diseases, have been reported in 22%–86% of patients with SSc, depending on detection methods used [108], and have been associated with a higher frequency of pulmonary fibrosis [109]. 

## 14. Serum Biomarkers of Pulmonary Hypertension in SSc-ILD

A detailed analysis of pulmonary hypertension markers in SSc is outside of the scope of this paper. Pulmonary hypertension in SSc (SSc-PH) can occur in the absence of interstitial lung involvement, in association with ILD and/or as a consequence of left-sided heart disease. Compared to patients with SSc-PH alone, those with SSc-ILD associated PH have a worse prognosis, with a recently reported 3-year survival of 71% versus 47%, respectively (*P* = 0.07) [110]. Indicators suggestive of the development of PH in the context of ILD include a disproportionate reduction in DLCO compared to lung volumes and development of hypoxia [111, 112]. Echocardiography is used as a screening tool, but in patients with ILD is plagued by low specificity [113]. Although a right-sided cardiac catheterisation remains the gold standard for the diagnosis of PH, the procedure is invasive and cannot be used routinely for screening/monitoring purposes. 

Among serum biomarkers, natriuretic peptides, including brain natriuretic peptide (BNP) and N-terminal pro-brain natriuretic peptide (NT-pro-BNP), released in response to ventricular stretch by cardiomyocytes, have been established as informative markers of RV dysfunction in PH [114, 115]. BNP was found to be a marker of poor prognosis in patients with chronic lung diseases [116]. In a study of 90 ILD patients by Corte et al., including 18 with CTD-ILD, serum BNP correlated with echocardiographic estimates of pulmonary pressures. Furthermore, a serum BNP ≥20 pmol*·*L^−1^ was associated with a 14-fold increased mortality compared to patients with BNP <4 pmol*·*L^−1^ [117]. However, natriuretic peptide levels only rise when there is strain to the right ventricle, and markers of the earlier stages of pulmonary vasculopathy are needed to allow early intervention. 

## 15. Conclusion

The majority of the studies performed to date have identified a link between serum proteins and the presence or severity of ILD in the context of SSc. In a disease with extremely variable presentation and outcome, there is an unmet clinical need for biomarkers predictive of ILD progression over time, independently of disease severity. In particular, clinicians need a biomarker to target patients with early and limited ILD at higher risk of disease progression for early therapy, so as to prevent the development of extensive disease. Ideally, a biomarker would also provide early assessment of response to treatment and could theoretically identify subsets of SSc-ILD with differential response to targeted therapies. In providing information on the likelihood of future ILD progression, biomarkers should also allow better selection of SSc-ILD patients for clinical trials, to enrich trial cohorts with patients more at risk of disease progression.

To date, there have been very few studies evaluating the ability of biomarkers to predict SSc-ILD progression. The most promising biomarkers thus far appear to be KL-6 and CCL18, and the evidence for their utilisation in predicting likelihood of progression of SSc-ILD and monitoring treatment response is encouraging. However, the panel of investigated biomarkers so far has been limited. In idiopathic pulmonary fibrosis, prospective, multicentre, longitudinal trials are underway to identify if a broad array of biological biomarkers collected at the time of diagnosis and at various longitudinal time points can be used to predict the disease course—COMET: correlating outcomes with biochemical markers to estimate time-progression in IPF (NCT01071707) and PROFILE: prospective observation of fibrosis in the lung clinical endpoints (NCT01110694). A similar endeavour is yet to be undertaken in SSc-ILD.

Prospective, well-designed studies, with detailed characterisation of ILD at baseline, to allow adjustment for disease severity and meticulous monitoring of disease progression outcomes and serum biomarkers are needed. The use of high throughput gene expression, protein and immune serum biomarker profiling is likely to identify a combination of biomarkers, each expressing different pathogenetic pathways, that when combined may provide a powerful indication of the likelihood of ILD progression and allow early evaluation of response to treatment.
